# Analysis of the Endoplasmic Reticulum Subproteome in the Livers of Type 2 Diabetic Mice

**DOI:** 10.3390/ijms131217230

**Published:** 2012-12-17

**Authors:** Edmond Changkyun Park, Gun-Hwa Kim, Sung-Ho Yun, Hye Li Lim, Yeonhee Hong, Sang-Oh Kwon, Joseph Kwon, Young-Ho Chung, Seung Il Kim

**Affiliations:** 1 Division of Life Science, Korea Basic Science Institute, Daejeon 305-806, Korea; E-Mails: edy525@kbsi.re.kr (E.C.P.); genekgh@kbsi.re.kr (G.-H.K.); sungho@kbsi.re.kr (S.-H.Y.); hyeli1984@kbsi.re.kr (H.L.L.); hyh1209@kbsi.re.kr (Y.H.); kso@kbsi.re.kr (S.-O.K.); 2 Pioneer Research Center for Protein Network Exploration, Korea Basic Science Institute, Daejeon 305-806, Korea; 3 Department of Functional Genomics, University of Science and Technology, Daejeon 305-350, Korea; 4 Gwangju Center, Korea Basic Science Institute, Gwangju 500-757, Korea; E-Mail: joseph@kbsi.re.kr; 5 Department of Bio-Analytical Science, University of Science and Technology, Daejeon 305-350, Korea

**Keywords:** type 2 diabetes, *db/db*, liver, endoplasmic reticulum, ER stress, proteomics

## Abstract

Type 2 diabetes is a chronic metabolic disease that results from insulin resistance in the liver, muscle, and adipose tissue and relative insulin deficiency. The endoplasmic reticulum (ER) plays a crucial role in the regulation of the cellular response to insulin. Recently, ER stress has been known to reduce the insulin sensitivity of the liver and lead to type 2 diabetes. However, detailed mechanisms of ER stress response that leads to type 2 diabetes remains unknown. To obtain a global view of ER function in type 2 diabetic liver and identify proteins that may be responsible for hepatic ER stress and insulin resistance, we performed proteomics analysis of mouse liver ER using nano UPLC-MS^E^. A total of 1584 proteins were identified in control C57 and type 2 diabetic *db/db* mice livers. Comparison of the rER and sER proteomes from normal mice showed that proteins involved in protein synthesis and metabolic process were enriched in the rER, while those associated with transport and cellular homeostasis were localized to the sER. In addition, proteins involved in protein folding and ER stress were found only in the rER. In the livers of *db/db* mice, however, the functions of the rER and sER were severely disrupted, including the capacity to resolve ER stress. These results provide new insight into the research on hepatic insulin resistance and type 2 diabetes and are suggestive of the potential use of the differentially expressed hepatic ER proteins as biomarkers for hepatic insulin resistance and type 2 diabetes.

## 1. Introduction

The endoplasmic reticulum (ER) is a central organelle that plays essential roles in cell survival and homeostasis. Morphologically, the ER is a continuous membrane-enclosed organelle that consists of functionally and structurally distinct domains that include the nuclear envelope (NE) and peripheral ER. The NE consists of a membrane bilayer with a lumen and surrounds the nucleus. The peripheral ER is a network of tubules and sheets spread throughout the cytoplasm and subdivided into the rough ER (rER) and the smooth ER (sER). The rER is studded with ribosomes while the sER is not [1]. These distinct subcompartments are known to have different functions. The rER participates in the synthesis and packaging of proteins, and the sER is important for the synthesis and metabolism of lipids and steroids, metabolism of carbohydrates, regulation of calcium concentration, and drug detoxification [2]. The difference in the relative abundance of rER and sER in various cell types correlates with their functions [3]. Disorders that affect ER function can lead to cell dysfunction and disease [4–6].

Diabetes mellitus is a chronic metabolic disorder characterized by elevated blood glucose levels (hyperglycemia) caused by decreased secretion of insulin, impaired insulin signaling, or both. Type 1 diabetes results from the excessive loss of pancreatic β-cells, while type 2 diabetes is the consequence of a progressive decline in β-cell function [7]. Type 2 diabetes is the most common form of diabetes, accounting for more than 90% of diabetic patients. Type 2 diabetes is characterized by two major features, such as insulin resistance of the liver, adipose tissue and muscle, and impaired insulin secretion from pancreatic β-cells [8]. Insulin resistance is caused by a variety of genetic and metabolic factors. The most common etiological factor is obesity [9].

The liver is essential organ for the regulation of fatty acid, lipoprotein and carbohydrate metabolism and plays a key role in glucose homeostasis. Hepatic insulin resistance is a common feature of type 1 and type 2 diabetes and contributes to diabetic hyperglycemia [10,11]. It has been known that hepatic insulin resistance is ascribed to hepatocellular lipid accumulation and inflammation. Recently, hepatic ER stress induced by obesity and/or metabolic stress has been suggested as a mechanism underlying the development of insulin resistance in the liver [12,13].

Numbers of proteomic studies have reported the differentially expression of proteins in type 2 diabetes [14]. In addition, hepatic proteomes were analyzed in insulin resistant and type 2 diabetic models [15–19]. The focus of these studies has been on a global understanding of the protein changes that occur in type 2 diabetes. Although ER stress is known to be a major risk factor of hepatic insulin resistance and type 2 diabetes, no attempts to examine the specific proteome changes that occur in the ER in type 2 diabetic livers have been reported. Recently, Song *et al.* optimized a method for the preparation of rER and sER from mouse livers [20] and elucidated the distinct function of each ER domain [21]. Here, we conducted a proteomic analysis of the hepatic ER subproteome in normal and type 2 diabetic mice using nano-UPLC-MS^E^. Comparison of the proteomic profile of hepatic ER proteins in normal and type 2 diabetic mice will contribute to a better understanding of the mechanisms and physiology of hepatic insulin resistance and type 2 diabetes, in addition to aiding in the identification of potential therapeutic targets to treat and/or prevent type 2 diabetes and associated hepatic complications.

## 2. Results

### 2.1. Preparation and Confirmation of ER Protein Fractions

**Figure 1:**
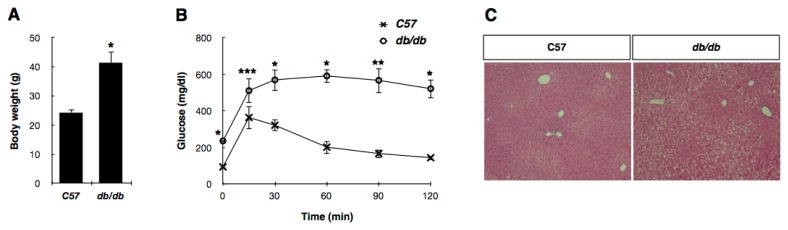
Physiological measurements in *db/db* mice. (**A**) Body weight of C57 control and *db/db* mice at 10 weeks; (**B**) Intraperitoneal glucose tolerance test (IPGTT) was performed by injecting 1 g/kg glucose intraperitoneally into indicated mice. Data are shown as means ± SD. *p*-Values were determined by Student’s *t*-test. ^*^p < 0.001, ^**^p < 0.005, and ^***^p < 0.05 *versus* C57 control mice; (**C**) Liver sections were prepared from C57BL/6J and *db/db* mice, and sections were stained with hematoxylin-eosin.

To investigate the hepatic subproteome of type 2 diabetes, *db/db* mice were used as a model of type 2 diabetes, and its lean littermates (C57BL/6J) were used as the control. The *db/db* mouse strain is a well known model of obesity, diabetes, and dyslipidemia, in which leptin receptor activity is deficient (Lepr^−/−^). The mutant mice begin to exhibit obesity at around three to four weeks of age and develop diabetes at approximately 10 weeks of age [22,23]. First, we measured physiological parameters to ensure that only diabetic mice were included in the experimental group. The body weights of the 10-week-old *db/db* mice were much higher than those of the C57 control mice (Figure 1A). The *db/db* mice had impaired glucose tolerance and significantly elevated blood glucose levels (Figure 1B), indicating that these mice were in an insulin resistant state. In addition, the livers from the *db/db* mice showed severe hepatic steatosis (Figure 1C).

**Figure 2:**
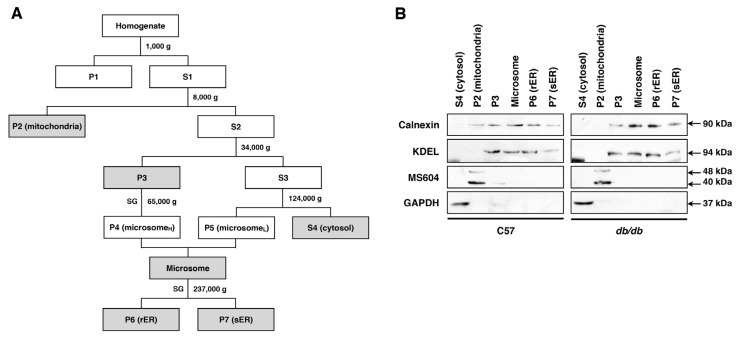
Subcellular fractionation of mouse liver. (**A**) Flow diagram for the subcelluar fractionation. P, pellet; S, supernatant; SG, sucrose gradient; (**B**) Immunoblot assessment of the subcellular fractionation. Calnexin and KDEL are ER markers, MS604 is a mitochondria marker, and GAPDH is a cytosol marker.

Liver tissues were isolated from 10-week-old *db/db* (*n* = 15) and C57 control (*n* = 15) mice, and six subcellular fractions, including the rER and sER, were obtained from the whole fresh liver tissues (Figure 2A). To confirm the purity of each fraction, the subcellular fractions were analyzed by organelle-specific protein antibodies: for the ER, anti-Calnexin and anti-KDEL, for the mitochondria, MS604, and for the cytoplasm, anti-GAPDH antibodies were used. Western blot results showed that fractions for the rER and sER fractions were cleanly and effectively separated from the other subcellular fractions (Figure 2B).

### 2.2. Identification and Quantification of the Hepatic ER Subproteome

To identify differentially expressed ER proteins between in normal and type 2 diabetic livers, ER subfractions were analyzed by LC-MS^E^ based protein identification. Compared to previous proteomic approaches, nano-UPLC-MS^E^ method based on label-free quantitative analysis enables large-scale comparison of all detectable proteins in complex samples separated by 1D-PAGE [24].

**Figure 3:**
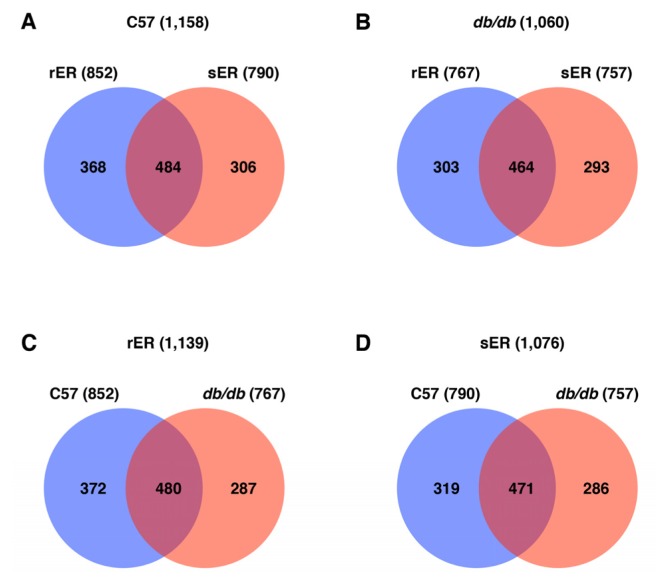
Venn diagrams showing the distribution of the unique ER proteins identified in C57 control and *db/db* mice livers. (**A**) Control mice livers; (**B**) *db/db* mice livers; (**C**) rER fraction; (**D**) sER fraction.

From the nano-UPLC-MS^E^ tandem MS of ER fractions, a total of 1584 proteins were identified. A list of identified proteins with quantitative data is provided in Table S1. Of the 1584 proteins, 1158 proteins (493 unique proteins) were identified in the ER of normal mice liver and 1060 proteins (415 unique proteins) were detected in the ER of *db/db* mice liver (Figure 3A,B). In the control mice, the rER expressed 852 proteins and the sER expressed 790 proteins. Of these, 484 (41.8%) proteins were commonly expressed between these ER subcompartments (Figure 3A). In type 2 diabetic mice, 767 proteins were expressed in the rER and 757 proteins were expressed in the sER. The hepatic ER subcompartments of type 2 diabetic mice showed common expression of 464 (43.8%) proteins (Figure 3B). It is of note that the total number of identified proteins expressed in the hepatic ER is fewer in type 2 diabetic mice than those in control mice. Interestingly, comparison of the rER and sER in control and type 2 diabetic mice revealed markedly different protein expression patterns. Only 480 out of 1139 proteins in the rER and 471 out of 1076 proteins in the sER were commonly expressed in control and type 2 diabetic mice livers (Figure 3C,D). This finding suggests that the protein profile of the ER in type 2 diabetic livers is markedly different from that of normal mice liver. In addition, the identified proteins that were differentially expressed in normal and type 2 diabetic ER would be good biomarkers for hepatic insulin resistance and type 2 diabetes.

Quantification analysis of the identified proteins showed that 493 and 415 unique proteins were expressed in C57 control and *db/db* mice liver, respectively (Tables 1 and S2). In the ER of normal mice livers, 113 out of 492 proteins (22.9%) were commonly expressed in the rER and sER, while the ER of diabetic mice livers shared 146 out of 415 proteins (35.1%) (protein list is shown in Tables S3 and S4). More importantly, the expression of the majority of proteins were changed in type 2 diabetic liver. The expression level of 364 out of 477 proteins (76.3%) and 371 out of 462 proteins (80.3%) were altered at least 1.5-fold level in the rER and sER, respectively (protein list is shown in Tables S5 and S6). This indicates that expression of ER proteins is largely changed in type 2 diabetes and suggests that normal ER function may be disrupted in the liver of type 2 diabetic mice.

### 2.3. ER Dysfunction in the Type 2 Diabetic Mouse Liver

**Figure 4:**
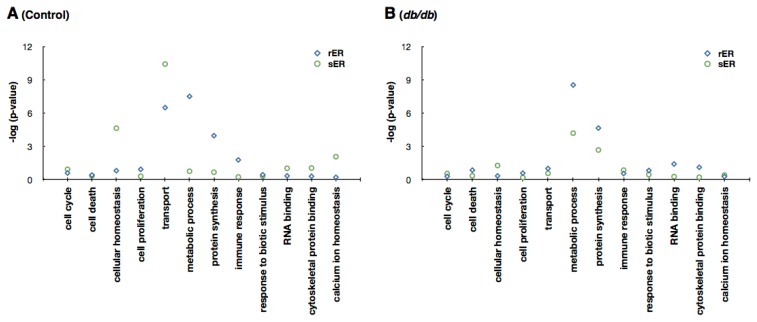
Functional distribution of proteins among unique proteins of the rER and sER using Gene Ontology (GO) annotation with the hypergeometric test. (**A**) C57 control mice livers; (**B**) *db/db* mice livers. Unique proteins showing that the expression levels are higher at least 1.5-fold in each fraction were used for analysis (for C57 rER, 214 proteins; C57 sER, 166 proteins; *db/db* rER 148 proteins; *db/db* sER, 121 proteins). Proteins showing that the expression is higher or lower at least 5-fold are considered as exclusively expressed proteins in the indicated fraction. The degree of enrichment or depletion of identified proteins in a given function category is represented as −log(*p*).

The difference in ER protein expression in normal and type 2 diabetic livers led to the examination of difference and state of ER function in type 2 diabetic mice livers. To examine the differences in protein expression profiles, the identified proteins were functionally categorized based on Gene Ontology (GO) annotation terms using GOfact. The results showed that in the livers of control mice, proteins involved in protein synthesis and metabolic process were significantly enriched in the rER, while proteins associated with transport and cellular homeostasis were significantly enriched in sER (Figure 4A). This coincides with the findings presented in previous reports [21]. In type 2 diabetic mice livers, however, no significant functional differences were observed between the rER and sER. Proteins involved in protein synthesis and metabolic process were identified in both ER subcompartments. Moreover, proteins responsible for distinct ER functions were not detected in either the rER or the sER (Figure 4B). These results demonstrate that normal ER function is disrupted and the functional difference in ER subcompartments is ambiguous in the type 2 diabetic mouse liver.

### 2.4. Sensing and Responding to ER Stress

**Figure 5:**
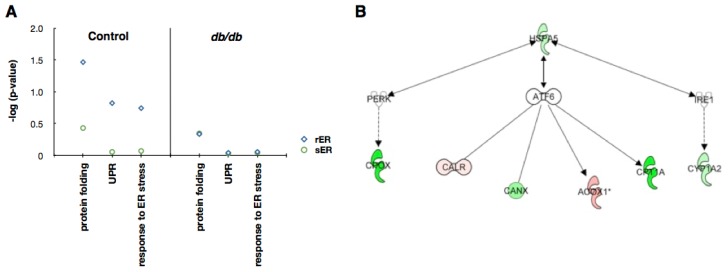
Proteins associated with ER stress. (**A**) Functional distribution of proteins involved in ER stress using Gene Ontology (GO) annotation with the hypergeometric test. Unique proteins showing that the expression levels are higher at least 1.5-fold in each fraction were used for analysis. The degree of enrichment or depletion of identified proteins in a given function category is represented as −log(*p*); (**B**) Protein network analysis. IPA-determined network of identified proteins in the rER that may be involved in ER stress signaling. Red, up-regulated in *db/db* mouse liver; green, down-regulated in *db/db* mouse liver.

Since hepatic ER dysfunction in type 2 diabetes is mainly caused by ER stress [25], we tried to identify which ER subcompartment is responsible for sensing and responding to metabolic ER stress. GO annotation clearly showed that in control mice, proteins associated with sensing and responding to ER stress were significantly enriched in the rER. In mice with type 2 diabetes, however, proteins associated with ER stress response were not expressed in the hepatic ER (Figure 5A). This suggests that the ER in type 2 diabetic livers may not be capable of re-establishing homeostasis.

To further identify putative proteins that could be associated with ER stress, we performed protein network analysis using proteins expressed exclusively in the rER. The result showed that seven proteins, such as HSPA5 (heat shock 70 kDa protein 5), CPOX (coproporphyrinogen oxidase), CALR (calreticulin), CANX (calnexin), ACOX1 (peroxisomal acyl-coenzyme A oxidase 1), CRT1A (calreticulin-1), and CYP1A2 (cytochrome P450 1A2), were related to three ER stress sensors: inositol-requiring enzyme 1 (IRE1), activating transcription factor 6 (ATF6) and PKR-like ER kinase (PERK) (Figure 5B). It suggests that these proteins could be used as potential biomarkers for ER stress and/or hepatic insulin resistance

## 3. Discussion

The ER is an essential organelle that functions in the quality control of newly synthesized proteins. The ER also has many additional functions, such as Ca^2+^ storage, and the synthesis of lipids and steroids [2]. Disruption of any of these ER functions can induce ER stress, which leads to cell dysfunction and contributes to disease progression [6]. In the liver, ER dysfunction induces hepatic insulin resistance, a primary cause of type 2 diabetes [12,26]. Here, we performed proteomic analysis to investigate changes that occur in the ER in the livers of type 2 diabetic mice. Using comparative analysis of ER subcompartments isolated from normal and type 2 diabetic mice livers, we demonstrated that the ER of type 2 diabetic mice livers is in a significantly altered state and that the most of the normal functions of the ER are disrupted.

The ER consists of rER and sER, the structurally and functionally distinct subcompartments [2]. The rER directs protein synthesis, while the sER functions in transport and calcium homeostasis (Figure 4A). When we evaluated the specialized functions of the rER and sER in the type 2 diabetic livers, we found that the diabetic sER contained proteins involved in protein synthesis, while proteins associated with transport and calcium homeostasis were not expressed (Figure 4B). This demonstrates that the rER and sER in the type 2 diabetic liver are not functionally distinguishable. In addition, each ER compartment demonstrated highly different proteome patterns. Normal and type 2 diabetic livers commonly expressed less than 45% of the total proteins identified in the rER or sER (Figure 3C,D). In the case of proteins with quantitative data, rER and sER share only about 35% proteins both in normal and type 2 diabetic mice (Table 1). Further functional annotation analysis of the ER proteome using the Ingenuity Pathway Analysis (IPA) tool clearly showed that each ER subcompartment performs significantly different functions (Figure S1). This also strongly supports our findings that the normal ER function is disrupted in the liver of type 2 diabetic mouse. Furthermore, the differentially expressed genes in the ER of normal and type 2 diabetic liver may serve as biomarkers for hepatic insulin resistance and type 2 diabetes.

Hepatic ER stress induces insulin resistance in liver tissue and eventually leads to type 2 diabetes [26]. ER stress triggers the evolutionarily conserved mechanism referred to as the unfolded protein response (UPR) pathway. Activation of UPR signaling leads to transcriptional activation of ER chaperones and reduced protein synthesis in an effort to re-establish homeostasis of ER functions [26]. We found that the total number of proteins expressed in the diabetic ER is 8.5% fewer than that in the ER of control mice. In the type 2 diabetic ER, 1060 proteins were identified, while 1158 proteins were identified in the normal ER (Figure 3). The difference of number of proteins expressed in the hepatic ER intensifies when we compare the number of proteins with quantitative data. The hepatic ER of type 2 diabetes consists of 15.8% fewer proteins than the ER of normal mice liver (Table 1). Therefore, it strongly suggests that the ER in the type 2 diabetic mouse liver undergoes severe ER stress and this leads to the attenuation of translation. Moreover, the expression of proteins involved in normal ER functions was suppressed in the ER of type 2 diabetic livers (Figure 4). This indicates that excessive ER stress under the condition of metabolic stress results in severe ER dysfunction.

ER stress triggers the UPR pathway, which involves IRE1, ATF6 and PERK. These ER stress sensors are localized in the ER membrane and transmit ER stress signals to the cytoplasm [4]. The UPR components play dual roles, acting as beneficial regulators under physiological conditions or as triggers of cellular dysfunction and apoptosis under situations of chronic stress. The initial combined activation of ATF6, IRE1 and PERK signaling generates a cytoprotective signal. Conversely, down-regulation of ATF6 and IRE1, coupled with maintenance of PERK activity, results in the induction of apoptotic cell death [27]. Since the function of UPR signaling has been focused, neither the initial process nor the localization of UPR signaling has been identified. In this study, we found that the rER contains proteins that are involved in protein folding, the UPR and the response to ER stress, while the sER does not (Figure 5A). These data may provide evidence that the sensing of and responding to ER stress occurs in the rER.

We also found that proteins associated with ER stress response are depleted in the ER of type 2 diabetic mice liver (Figure 5A). Previous reports also revealed the deficient ER stress response in *db/db* mice liver. The expression of ATF6 is drastically suppressed in the liver of *db/db* mice [28]. In contrast, the expression of PERK is highly upregulated in *db/db* mice liver [29]. In addition, Morand *et al*. showed that hepatic ER proteins ER60, ERp46, and ERp29, which are known to be associated with protein folding, were down-regulated in insulin-resistant hamsters [30]. These suggest that the ER of type 2 diabetic liver may not be capable of overcoming ER stress and undergo apoptotic cell death.

From the protein network analysis, we identified seven putative proteins (HSPA5, CPOX, CALR, CANX, ACOX1, CRT1A, and CYP1A2) that could be functionally associated with ER stress (Figure 5B). HSPA5 (heat shock 70 kDa protein 5), is a member of heat shock protein 70 family and also known as glucose-regulated protein 78 kDa (GRP78/BiP). It is localized in the lumen of ER and is involved in the protein folding and assembly. As this protein interacts with many ER proteins including ER sensors, it may play a key role in monitoring ER stress and protein transport through the cell [31,32]. It is also well known that its expression is decreased in liver of diabetic mice [33]. CPOX (coproporphyrinogen oxidase) is the sixth enzyme in heme biosynthetic pathway. It catalyzes the two-step oxidative decarboxylation of coproporphyrinogen III to coproporphyrinogen IX, a precursor of heme [34]. CALR (calreticulin), CRT1A (calreticulin-1), and CANX (calnexin) are molecular chaperones that act as major Ca^2+^ binding/storage proteins in the lumen of the ER. They are also involved in the quality control of newly synthesized proteins and glycoproteins, interacting with various other ER chaperones [35–37]. CALR is also found in the nucleus, cytoplasm, cell membrane, and extracellular matrix suggesting that it may have a role in transcription regulation and cell adhesion [35]. ACOX1 (peroxisomal acyl-coenzyme A oxidase 1) is the first enzyme of the peroxisomal fatty acid β-oxidation pathway, which catalyzes the desaturation of acyl-CoAs to 2-*trans*-enoyl-CoAs [38]. Fatty acid β-oxidation is closely associated with ER stress and type 2 diabetes [38,39]. CYP1A2 (cytochrome P450 1A2) is a member of cytochrome P450 superfamily of enzyme and is involved in the metabolism of xenobiotics in the ER [40]. Recently, studies report that the expression of CYPs are dynamically regulated in the diabetic liver [41–43]. Further studies are needed to determine whether any of the proteins could be responsible for inducing or transmitting ER stress signaling and eventually cause insulin resistance and type 2 diabetes.

## 4. Experimental Section

### 4.1. Animals and Tissue Preparation

Male C57BL6/J and C57BL/KsJ-*db/db* mice were purchased from Central Lab. Animal Inc. (Seoul, Korea) at the age of 8 weeks and housed in laboratory cages under a 12 h dark/light cycle for 2 weeks of acclimatization. Intraperitoneal glucose tolerance test (IPGTT) was performed after overnight fasting by intraperitoneal injection of 1 g/kg glucose dissolved in PBS. Blood glucose concentrations were determined using a One Touch Ultra glucometer (LifeScan, Milpitas, CA, USA) before (0 min) and 15, 30, 60, 90 and 120 min after glucose injection. The mice were starved for 24 h before sacrifice. Blood was collected from orbital sinus at the time of sacrifice. Fifteen mice of each group were sacrificed by spinal dislocation and whole livers were rapidly excised and immersed in cold 0.9% NaCl for completely remove remained blood. Small fractions of isolated livers were fixed in 10% neutral buffered formalin solution, and paraffin sections were stained with hematoxylin-eosin.

### 4.2. Separation and Fractionation of Organelles of Mouse Liver

The fresh liver tissues (*n* = 15) were cut into large pieces and homogenized with dispenser in 5 volume of their weight (mL/g) of cold homogenization buffer (0.25 M sucrose, 10 mM HEPES pH 7.5, protein inhibitor cocktail). The homogenate was gravity-filtered with two layers of 100-mesh nylon cloth (100 μm, BD Bioscience, Franklin Lakes, NJ, USA) and centrifuged (1000 rpm, 15 min, 4 °C) for removing debris.

ER proteins were purified according to the procedure previously described [20] with minor modification. The illustrated protocol for subcellular fractionation is shown in Figure 2A. All procedures were operated at 4 °C. In brief, the homogenate was centrifuged at 1000× *g* for 10 min. The pellet 1 (P1) was discarded and supernatant 1 (S1) was collected for centrifugation at 8000× *g* for 25 min. The pellet 2 (P2) was used for purifying mitochondria by nycodenz gradient centrifugation. The supernatant 2 (S2) was centrifuged at 34,000× *g* for 30 min. Then the supernatant 3 (S3) was centrifuged at 124,000× *g* for 60 min. The supernatant 4 (S4) is used for cytosolic fraction and the pellet (P5) is collected for light micosomes. The pellet 3 (P3) was used for purifying heavy microsomes (P4) by sucrose/CsCl gradient centrifugation. After that P4 and P5 were combined for microsome fraction. The microsome fraction was centrifuged at 237,000× *g* for 120 min through sucrose/CsCl gradient tube to separate rER and sER. The pellet 6 (P6) is used for rER and the interface was diluted and centrifuged at 124,000× *g* for 60 min to collect sER pellet (P7). All the fractions were snap frozen in liquid nitrogen and stored at −80 °C.

### 4.3. SDS-PAGE and In-Gel Digestion

100 μg protein of each fraction were separated by 12% SDS-PAGE. The gels were stained by Coomassie blue and divided into ten fragments according to molecular weight. The gel fragments were desained in 50% acetonitrile and 10 mM ammonium bicarbonate and rinsed with 100% acetonitrile. After drying with a speed vacuum concentrator, the gels were incubated in a solution containing 10 mM DTT and 100 mM ammonium bicarbonate at 56 °C to reduce protein disulfide bonds. Same volume of 55 mM iodoacetamide and 100 mM ammonium bicarbonate were added for alkylate cysteines in dark condition. The gels were then washed out with three volumes of distilled water and dried using the speed vacuum concentrator. After swelling gels with 300 μL of 50 mM ammonium bicarbonate, proteins were digested with 15 μL trypsin (0.1 μg/μL) at 37 °C for 18 h. Then the digested peptides were recovered twice with a solution containing 50 mM ammonium bicarbonate, 50% acetonitrile, and 5% trifluoroacetic acid (TFA). The resulting peptide extracts were pooled, lyophilized in a vacuum centrifuge, and dissolved in 0.5% TFA solution prior to MS or MS/MS analysis.

### 4.4. Nano-UPLC-MS^E^ Tandem Mass Spectrometry and Database Search

For nano-LC and tandem MS analysis, a nano-ACQUITY Ultra Performance LC Chromatography™ equipped Synapt™ HDMS System (Waters Corporation, Milford, MA, USA) was used as described previously [44]. For the elimination of salts, the peptide mixture was loaded on a 180 μm × 20 mm Symmetry C_18_ RP 5 μm enrichment column. The flow-through peptides were directly appled on a 180 μm × 250 mm nano-ACQUITY UPLC 1.7 μm BEH300 C_18_ RP column Tryptic digested peptides (5 μL) were loaded onto the enrichment column with mobile pahse A contained 3% acetonitrile in water with 0.1% formic acid. A step gradient was used at flow rate of 300 nL/min. This included a 3%–40% mobile phase B, 97% acetonitrile in water with 0.1% formic acid, over 95 min, 40%–70% mobile phase B over 20 min, followed by a sharp increase to 80% B in within 10 min. During the data acquisition, collision energy of low energy MS mode and elevated energy mode (MS^E^) were set to 4 eV and 15–40 eV energy ramping, respectively.

Protein identification was carried out with the continuum LC-MS^E^ data using PLGS (ProteinLynx GlobalServer) version 2.3.3 (Waters Corporation, Milford, MA, USA). Protein identification was set up with IPI mouse database (version 3.44) Criteria of search condition by PLGS were applied with a peptide tolerance of 100 ppm; fragment tolerance of 0.2 Da; missed cleavage of 1; and Cystein, carbamidomethylation at C. Analysis of quantitative changes with a confidence of >95% in protein abundance, which was based on measurements of peptide ion peak intensities observed in low collision energy mode (MS) in a triplicate set, was carried out using Expression™ Software (version 2).

### 4.5. Bioinformatic Analysis

All of the identified proteins were subjected to query functional annotation analysis and direct/indirect interactions on proteins that are involved in pathways associated with ER stress using Ingenuity Pathway Analysis (IPA) tool [45]. For functional enrichment analysis of Gene Ontology (GO) categories, we used GOfact online tool [46].

### 4.6. Western Blot

Western blot was performed according to standard protocol as previously described [47]. The protein blot was detected using a luminescent image analyzer LAS-4000 mini system and software (FujiFilm). The following antibodies were used: rabbit anti-calnexin (PAB15419, Abnova, Taiwan), mouse anti-KDEL (ADI-SPA-827-D, Enzo Life Science, Plymouth Meeting, PA, USA), rabbit anti-GAPDH (sc-35778, Santa Cruz Biotechnology, Santa Cruz, CA, USA), Mitosciences-MS604 (ab110413, Abcam, Cambridge, UK). Antibodies were used at dilution of 1:2000.

## 5. Conclusions

In summary, comparative analysis of the hepatic ER subproteome revealed that the normal function of the ER is disrupted in type 2 diabetes, rendering the ER incapable of re-establishing homeostasis. Further studies are needed to determine whether any of the proteins described herein could be responsible for ER dysfunction. Identifying the mechanisms that cause ER dysfunction will provide us with clues to preventing or curing the insulin resistance caused by ER stress.

## Figures and Tables

**Table 1:** Summary of quantitative analysis of ER proteome from normal and *db/db* mice.

Ratio	C57 (rER/sER)		*db/db* (rER/sER)		rER (db/db/C57)		sER (*db/db*/C57)	
			
	T_Pro *	U_Pro **	T_Pro	U_Pro	T_Pro	U_Pro	T_Pro	U_Pro
Ratio ≥ 5	360	174	251	118	269	133	276	143
5 > Ratio ≥ 1.5	102	40	69	30	122	40	108	42
1.5 > Ratio > 0.67	319	113	362	146	282	113	297	91
0.2 < Ratio ≤ 0.67	63	23	33	15	76	26	66	27
Ratio ≤ 0.2	301	143	221	106	325	165	313	159
Subtotal	1145	493	936	415	1074	477	1060	462
Total (T_Pro/U_Pro)	1584/685							

* T_Pro, total identified protein;

** U_Pro, unique protein (Overlapping proteins were eliminated).
